# New Insights on Solvent-Induced Changes in Refractivity and Specific Rotation of Poly(propylene oxide) Systems Extracted from Channeled Spectra

**DOI:** 10.3390/ijms25094682

**Published:** 2024-04-25

**Authors:** Alexandru Zara, Raluca Marinica Albu, Iuliana Stoica, Andreea Irina Barzic, Dan Gheorghe Dimitriu, Dana Ortansa Dorohoi

**Affiliations:** 1 Faculty of Physics, “Alexandru Ioan Cuza” University, 700506 Iasi, Romania; zara.alexandru@gmail.com (A.Z.); ddorohoi@uaic.ro (D.O.D.); 2 “Petru Poni” Institute of Macromolecular Chemistry, 700487 Iasi, Romania; albu.raluca@icmpp.ro (R.M.A.); stoica_iuliana@icmpp.ro (I.S.);

**Keywords:** chiral polymer, solvent influence, refractivity, circular birefringence, specific rotation, channeled spectra

## Abstract

Investigation of chiroptical polymers in the solution phase is paramount for designing supramolecular architectures for photonic or biomedical devices. This work is devoted to the case study of poly(propylene oxide) (PPO) optical activity in several solvents: benzonitrile, carbon disulfide, chloroform, ethyl acetate, and *p*-dioxane. To attain information on the interactions in these systems, rheological testing was undertaken, showing distinct variations of the rheological parameters as a function of the solvent type. These aspects are also reflected in the refractive index dispersive behavior, from which linear and non-linear optical properties are extracted. To determine the circular birefringence and specific rotation of the PPO solutions, the alternative method of the channeled spectra was employed. The spectral data were correlated with the molecular modeling of the PPO structural unit in the selected solvents. Density functional theory (DFT) computational data indicated that the torsional potential energy—related to the O1-C2-C3-O4 dihedral angle from the polymer repeating unit—was hindered in solvation environments characterized by high polarity and the ability to interact via hydrogen bonding. This was in agreement with the optical characterization of the samples, which indicated a lower circular birefringence and specific rotation for the solutions of PPO in ethyl acetate and *p*-dioxane. Also, the shape of optical rotatory dispersion curves was slightly modified for PPO in these solvents compared with the other ones.

## 1. Introduction

Most natural macromolecular compounds display an intriguing structural feature, known as chirality or handedness [1,2]. The latter denotes that it is not possible to superimpose a chemical structure on its mirror picture, even if rotation or translation movements are applied. This aspect has tremendous importance in a variety of biological and physiological processes [3]. Chiral materials have attracted the scientific community’s attention to elucidate the involvement of nature’s refined self-assembly procedures to create new functional architectures with unique properties [4,5]. After many efforts, it was demonstrated that the chirality of polymers can be generated by stereogenic centers in the backbone or the chirality of the peculiar segments (i.e., nucleotides). In other cases, it was found that certain macromolecules exhibit chiral architectures as a result of induced conformation of the chains, which are built based on achiral subunits [5].

A prevalently encountered chiral architecture is the helical one, and the sense of helicity may be tuned in several ways. At the molecular level, the chiral structure of macromolecular compounds exists in two principal forms, namely configurational and conformational chirality. The first one refers to asymmetric spatial arrangement of atoms/groups in the chains [6]. It is worth mentioning that, in this situation, the length and the dimension of the substituents are responsible for potential racemization. Thus, it is essential to monitor the stereoregularity of all macromolecular segments. The changes in the configuration entail the breaking and reorientation of the covalent bonds to produce new ones, resulting in products with similar molecular structures but distinct stereoregularities [4]. Regarding the conformational chirality, transitions can be easily attained when rotations of single bonds occur, modifying the spatial disposition of chemical groups [4]. Based on these findings, tailoring the handedness by adapting the chemical structure of natural polymers [7] or by preparation of synthetic polymers [8] was attempted. Both categories of materials have practical implications in biomedicine [9], catalysis [10], pharmaceutics [11], and optoelectronics [12].

A particular interest was given to the study of optical radiation interaction with chiral polymers to emphasize the factors that affect the optical activity in connection with the compound’s stereochemistry [13]. Some reports show that the optical rotation is influenced not only by the chemical structure but also by the insertion of a salt additive in the polymer solution [14,15], pH [16], temperature [17,18], and the solvent type and mixture [19,20,21]. Concerning the solvent’s implications in the optical activity, it was proved that the solvent’s nature has effects on the interactions in the system and also on the conformation (via changes in some dihedral angles) [19,22]. Deep knowledge of these aspects is beneficial for designing novel materials with tailored optical activity.

Generally, the optical activity is experimentally determined by means of polarimetry, which involves the travelling of polarized light through the examined sample and evaluates the rotation angle of the vibration plane of the radiation electric field upon exiting the chiral medium. Most polarimeters enable experiments at a fixed wavelength, while the devices that allow for the evaluation of the optical rotation dispersion (ORD) are more expensive. An alternative procedure to determine ORD was recently developed, relying on the channeled spectrum [23,24,25]. The latter is defined as a spectrum where the intensity presents a periodic modulation function of the wavenumber and is a useful tool for examination of the dispersion properties [26,27]. Thus, the spectrum channel can be regarded as being the region of the spectrum between two successive minima. For such measurements, two polarizers are introduced in the measurement beam of the spectrophotometer and the cell with the studied solution placed between them, while in the compensatory beam, two other polarization filters are inserted [24,28]. When exiting the anisotropic medium, the projection of the electric field intensity can be described via an expression relating the circular birefringence (Δ*n*), concentration, and thickness of the sample. The radiation emerging from the chiral polymer solution presents components with various degrees of rotation. By depicting the minima and maxima of the emergent flux density (based on recorded channeled spectra) as a function of the wavelength, it is possible to determine the birefringence and its dispersion parameter (*δ*). Since the specific rotation ([*θ*]) is linked to birefringence, it is very easy to compute the optical rotation at various wavelengths.

Among the optically active polymers, poly(propylene oxide) (PPO) is a synthetic polymer that displays alternating hydrophobic and hydrophilic units in its chains [29]. Thus, PPO is characterized by good solubility in many solvents. Also, this macromolecular material lacks toxicity, being used in the preparation of cosmetics, food products, excipients for drugs, and components for non-linear optics or photonics [30,31]. In a previous work [32], the ORD of PPO was measured in a benzene medium using channeled spectra technique, and it was found that the specific rotation decreased as the light wavelength became higher.

This paper contributes to previous efforts [32] by examining the solvent-induced effects on the ORD of PPO solutions in benzonitrile, carbon disulfide, chloroform, ethyl acetate, and *p*-dioxane. A deeper investigation was conducted to acquire a better comprehension of the impact of solvent features on variations in PPO optical behavior. Rheological testing was performed to capture information on the correlation among the shear response and PPO–solvent interactions. Density functional theory (DFT) computations performed on the PPO structural unit in the presence of solvent molecules revealed the possible formation of hydrogen bonding depending on the solvent type. The latter also affected the refractive index dispersive properties; thus, the linear and non-linear optical parameters of PPO can be tailored by proper selection of the solvation medium. Further, the channeled spectrum approach was applied to the prepared systems to gain new insights into the solvent’s implications on the PPO circular birefringence, its dispersion parameter, and specific rotation at various wavelengths. It was found that polymer–solvent interactions produce significant modifications in the aspect of channeled spectra, including on the ORD curves. This was also supported by the DFT studies on the torsional potential energy for the O1-C2-C3-O4 dihedral angle in PPO, the magnitude of which was smaller when using polar solvents. Such correlation among molecular modeling and rheological and optical data has not yet been reported, and it brings novel insights on the role of the chiral polymer–solvent compatibility on tailoring rheological and optical properties of PPO. Another original aspect of the present paper resides in determining the circular birefringence and optical rotation dispersion curves by an alternative new method involving the channeled spectrum. These new findings contribute to comprehension of the solution behavior of chiral polymer structures in environments having a distinct polarity and variable hydrogen bonding ability in relation to the material optical performance. This lies at the basis of the careful design of macromolecular architectures of practical importance in photonics and biomedical devices.

## 2. Results and Discussion

### 2.1. Rheological Behavior

**Figure 1:**
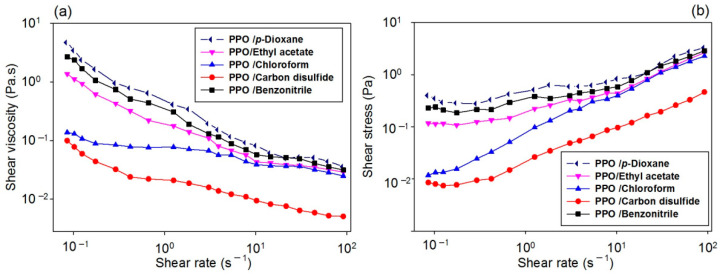
Dependence of (**a**) shear viscosity and (**b**) shear stress on shear rate for PPO solutions in several solvents.

Rheological testing of the PPO solutions was carried out to probe their flow behavior under imposed stress. Figure 1a depicts the shear viscosity against the shear rate for the PPO systems. The studied samples displayed a pseudoplastic behavior, and no zero-shear viscosity plateau was recorded, indicating that under shear deformation, the PPO chains aligned. It was found that the sample resistance to flow was different as a function of the solvent peculiarities. At low shear rates, the PPO solutions’ viscosity was affected by intermingling effects of solvent viscosity and solubility peculiarities (i.e., polarity and hydrogen bonding ability). As a general tendency, less viscous and low polar solvation media resulted in lower solution viscosity. Further information was extracted from the dependence of the shear stress versus shear rate (shown in Figure 1b), which can be fitted with the power law relation (1):

(1) $$\sigma =K_{0}\cdot {\dot{\gamma }}^{n_{flow}},$$

where *σ* is the shear stress, $\dot{\gamma }$ is the shear rate, *n_flow_* is the flow behavior index, and *K*_0_ is the solution consistency index.

By linearization of Equation (1), it is possible to evaluate the flow behavior index and solution consistency index of each PPO system. The attained values for *n_flow_* and *K*_0_ are listed in Table 1. The deviation from unitary values of the flow behavior index denotes that the analyzed fluid had no Newtonian character and, instead, it presented shear thinning flow properties (the viscosity was dependent on the applied deformation). Hence, the PPO solutions in benzonitrile, ethyl acetate, and *p*-dioxane presented values of *n_flow_* under 0.5, meaning that the viscosity had a steeper variation with the imposed shear rate. On the other hand, the PPO samples in chloroform and carbon disulfide displayed higher values for the *n_flow_* parameter. This was probably due to the smaller size of such low polar solvent molecules, which determine less polymer uncoiling, so that the chain orientation during shearing was reduced, and hence, the sample viscosity changed less abruptly upon shearing. This means that when specific molecular interactions take place between solute and solvent, the PPO chains are better penetrated by the polar molecules (benzonitrile, *p*-dioxane, or ethyl acetate), and they have a higher ability to uncoil and align under imposed deformation. The formation of specific interactions, like hydrogen bonding, is reflected in lowest *n_flow_* values because the macromolecular coils suffered more extensive unwinding, which enabled stronger orientation in the shear field. The specific molecular interactions that may or may not occur in the PPO samples are additionally reflected in the distinct values of the consistency index. As noted in Table 1, *K*_0_ varied as a function of the mixed effects of solvent viscosity and its ability to interact with PPO. When the polymer interacted with the solvent via specific interactions, the sample consistency was higher (e.g., PPO/*p*-dioxane), while in the case of weaker PPO–solvent interactions, a lower consistency index of the sample was observed (e.g., PPO/carbon disulfide).

**Figure 2:**
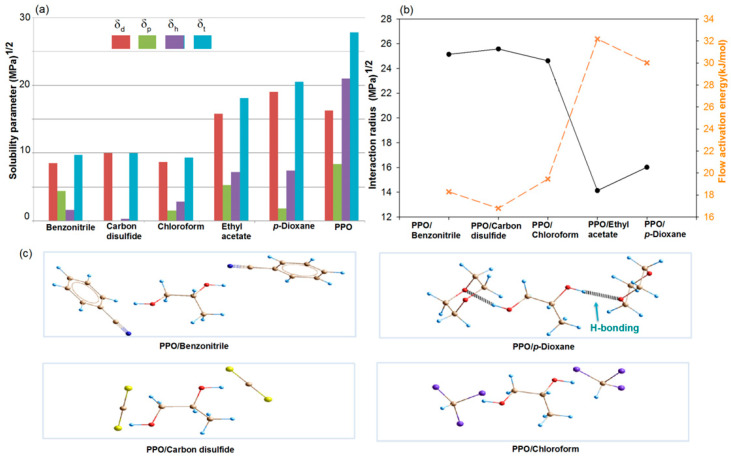
Solubility parameter components for the used solvents and those calculated for PPO (**a**), variation of the interaction radius (continuous line) and flow activation energy (dash line) (**b**) for all studied PPO solutions, and (**c**) interaction representation of the PPO structural unit in the presence of two solvent molecules (dashed line shows the hydrogen bonding between the PPO and the solvent).

A deeper comprehension of the solvent’s effects can be acquired by accounting for the solubility parameter. The dispersive (*δ_d_*), polar (*δ_p_*), and hydrogen bonding (*δ_h_*) components of the Hansen solubility parameter (HSP) for the chosen solvents were taken from the literature [33] and are plotted in Figure 2a in comparison to the solubility features of the PPO.

In the case of the investigated polymer, HSP values were obtained with the help of the new group-contribution approach developed by Stefanis and Panayiotou [34]. For the case where the total solubility parameter (*δ_t_*) values were closer, it can be stated that the solvent and polymer were more compatible, as observed for ethyl acetate and *p*-dioxane in regard to PPO. Based on these data, the dissolvability of the PPO in the analyzed solvents can be assessed using Equation (2):

(2) $$R_{0}=\sqrt{4{\left(\delta_{d,s}-\delta_{d,p}\right)}^{2}+{\left(\delta_{p,s}-\delta_{p,p}\right)}^{2}+{\left(\delta_{h,s}-\delta_{h,p}\right)}^{2}},$$

where *R*_0_ is the interaction solute/solvent radius, while the low indices “*s*” and “*p*” of the HSP components refer to the solvent and polymer.

The changes in magnitude of the interaction radius for each PPO/solvent system are presented in Figure 2b. The results indicate that the solubility distance was higher for PPO in carbon disulfide, benzonitrile, and chloroform, while this parameter was lower for PPO in ethyl acetate and *p*-dioxane. The latter solvents exhibited a polar character, which when combined with hydrogen bonding ability, led to a better interaction with PPO (Figure 2a,b). Also, polymer–solvent interactions were evaluated via the flow activation energy, which was determined by fitting the viscosity data (at shear rates close to zero) at several temperatures with the Arrhenius equation (3):

(3) $$\ln\eta =C+\frac{E_{a}}{RT},$$

where *C* is a constant, *E_a_* is the flow activation energy, *R* is the universal gas constant, and *T* is the absolute temperature.

The flow activation energy denotes the energy demanded by the molecules to acquire mobility against the frictional forces of the adjacent ones. When PPO was dissolved in solvents with low polarity (carbon disulfide, chloroform, benzonitrile), the interactions in such systems were weaker, and this was reflected in smaller values of the *E_a_* parameter (Figure 2b). Conversely, the magnitude of *E_a_* was higher for the PPO solutions in polar solvents with a hydrogen bonding ability (like *p*-dioxane and ethyl acetate). This is because when solute–solvent interactions are more powerful, a higher restrictive force exerted by the macromolecules must be overcome for the solution to flow. This is supported by literature data [35,36] and molecular modeling computations for studied PPO structural unit in the presence of two solvent molecules, as shown in Figure 2c.

Figure 2c depicts an illustrative scheme based on computational data, revealing the sites from the PPO structure that are available for the formation of the hydrogen bonds with certain solvent molecules. The optimized 3D molecular structures reflect the most stable geometries, having an essential role in the magnitude of the optical rotation. It is expected that the nature of the selected solvent affects the PPO repeating unit conformation as well as the torsion angle or potentially the length of specific bonds. According to the performed simulations, the PPO structural unit prevalently formed hydrogen bonds with the solvents having larger values of *δ_h_* (e.g., *p*-dioxane). When the PPO structural unit was under the influence of a non-polar solvent (e.g., carbon disulfide), the solute–solvent interactions were weak. This is supported by the literature [37], which explains that high solvent polarity is responsible for raising the rotation barrier of certain covalent bonds. In a polar solvent, the cohesion energy of the solute–solvent system is higher (stronger intermolecular forces), so that the overall mobility of the solute molecules in such a medium is diminished (bigger resistance to torsional deformation). Hence, when the PPO structural unit is placed in a polar solvent with a high probability to form hydrogen bonding (*p*-dioxan, ethyl acetate), the free rotation of certain substituents from the asymmetric carbon of the PPO structural unit is constrained, and a higher rotational barrier must be overcome to adopt different molecular conformations. The literature [38,39] states that the greater mobility of the substituents of the stereogenic centers leads to a wider range of molecular conformation, and each of them contributes to the optical rotation. Moreover, the literature [38] indicates that the solvent’s nature has a distinct influence on each substituent from a chiral molecule. For instance, polar substances display powerful intermolecular interactions with each other, producing nonsignificant changes in the contact radius of the substituents. The latter aspect is correlated with small values of the optical rotation [38]. It can be assessed that, when optical radiation passes through the PPO solutions in polar solvents, the changes in the rotation angle of the light polarization plane are limited compared to those of the PPO in less polar media. In addition, the solvent molecules having a similar ability to connect via hydrogen bonds with the chiral molecule produce distinct effects on the conformation and consequently on the optical activity [22]. This aspect is also reflected in optical anisotropy of the samples since the PPO contains an asymmetrical carbon atom and a chiral plane, which can be twisted as a function of the solvation environment’s polarity and hydrogen bonding ability. The literature [40] on molecular modeling of PPO in other solvents reveals that the conformation of this polymer is gauche-like in polar solvents (water and methanol), and a preference for *trans* conformation is prevalent in non-polar solvents (carbon tetrachloride and n-heptane) as a result of specific interactions via hydrogen bonding.

**Figure 3:**
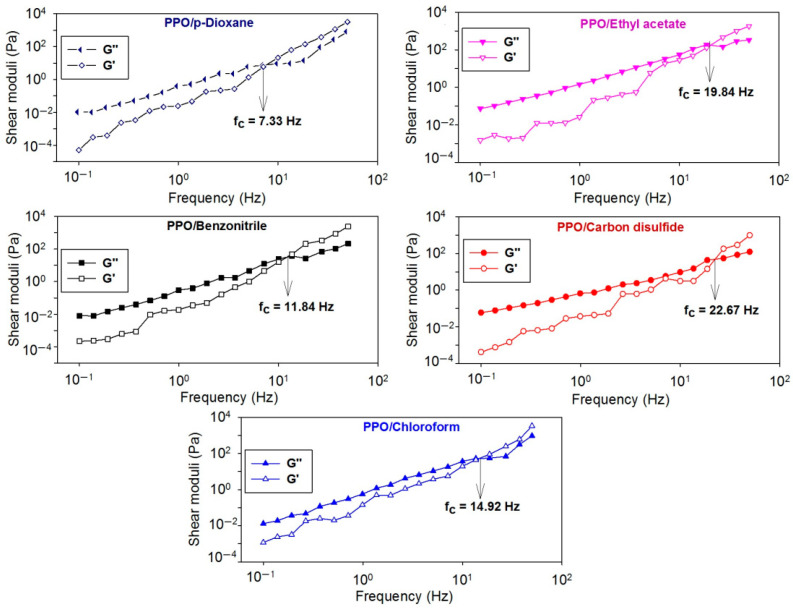
Shear oscillatory tests of the PPO solutions in several solvents.

The rheological analyses were continued by performing shear oscillatory experiments, and the obtained data are plotted in Figure 3. A frequency sweep of all samples revealed that at low frequencies, the loss (G″) modulus was higher in comparison with the storage (G′) one. Thus, the frequency (*f*) response of the rheological moduli was different, namely, G′~*f*^2^ and G″~*f*^1^. Also, no plateau appearing in the storage modulus curves was noticed, indicating a typical rheological behavior for the viscoelastic fluids [41]. As the frequency further increased, they became equal at a specific frequency, and then, the elastic component overcame the viscous one. The crossover frequency (*f_c_*) varied for the PPO solutions as a function of the solvent in the following order: *p*-dioxane < benzonitrile < chloroform < ethyl acetate < carbon disulfide. This means that more viscous PPO solutions will require a larger relaxation time and, as a result, the rheological moduli will become equal at a smaller crossover frequency. Thus, the balance between the viscous and elastic characteristics of the PPO samples is affected by the type of solvent. Another rheological report [42] on the shear oscillatory properties of PPO revealed that the distinct behavior of this polymer was associated with the polysiloxane cross-linker. Thus, in this case, the elastic modulus became larger than the viscous one for the entire shear frequency domain.

### 2.2. Linear and Non-Linear Optical Parameters

**Figure 4:**
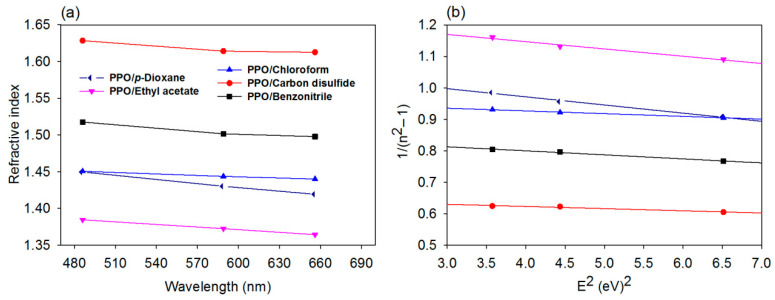
Refractive index dispersion (**a**) and 1/(n^2^ − 1) against the square photon energy (*E^2^*) (**b**) for the PPO solutions in several solvents.

The refractivity of a polymer solution can be regarded as a good indicator of the light speed and deflection characteristics upon traveling through it. The refractive index (*n*) of each PPO system was measured at different wavelengths, as illustrated in Figure 4a. The results prove that the samples exhibit normal dispersion curves. Regardless of the incident light wavelength, the refractive index magnitude of the PPO solutions changed as a function of the solvent, as follows: carbon disulfide > benzonitrile > chloroform > *p*-dioxane > ethyl acetate. This means that more sudden changes in the direction of light waves in regard to their orientation in the incident medium occurred in PPO/carbon disulfide and less in PPO/ethyl acetate. The refractive index dispersion of PPO and PPO solutions has not been published yet. However, for PPO, a refractive index of 1.457 has been reported [43].

From the dispersion data, additional information can be obtained by applying the theory of Wemple and DiDomenico [44], expressed through the relation (4):

(4) $$n^{2}=1+\frac{E_{d}E_{0}}{E_{0}^{2}-E^{2}},$$

where *E* is the photon energy, *E*_0_ refers to the average excitation energy for electronic transitions, and *E_d_* refers to the dispersion energy.

By graphical representation of 1/(n^2^ − 1) as a function of the square photon energy, as presented in Figure 4b, and fitting with a linear function, the values of *E_0_* and *E_d_* were obtained. The results are summarized in Table 2.

These linear optical parameters of PPO denote the influence of the solvent’s refractive features. The data indicate that dispersion energy, which is linked to the medium potency of interband photosensitive transitions, was smaller for PPO in *p*-dioxane and ethyl acetate due to their higher polar and hydrogen bonding features. Moreover, the single-oscillator energy for electronic transitions had lower values for these systems and therefore smaller band gap energy values compared with the case of PPO in less polar solvents.

Starting from *E_d_* and *E*_0_ data, it was possible to evaluate the static refractive index (*n*_0_) at zero photon energy, as depicted in relation (5):

(5) $$n_{0}={\left(1+\frac{E_{d}}{E_{0}}\right)}^{0.5}.$$

The results for this parameter are also included in Table 2, showing that solvents like chloroform, carbon disulfide, and benzonitrile led to higher values of *n_0_*, while for the polar solvents, this parameter had slightly smaller values.

Optical dispersion data also enabled the estimation of certain non-linear optical properties [45], such as first- and third-order optical susceptibilities (*χ*^(1)^ and *χ*^(3)^) and the non-linear refractive index (*n_nl_*), as shown in relations (6)–(8):

(6) $$\chi^{\left(1\right)}=\frac{E_{d}/E_{0}}{4\pi },$$

(7) $$\chi^{\left(3\right)}=6.82\cdot 10^{-15}\cdot {\left(E_{d}/E_{o}\right)}^{4}\;,$$

(8) $$n_{nl}=\frac{12\pi \chi^{\left(3\right)}}{n_{0}}.$$

All the results concerning the non-linear optical properties are listed in Table 2.

It was found that the values of the *χ*^(1)^ parameter were slightly smaller for the polymer solutions in *p*-dioxane and ethyl acetate in regard to the other studied systems, whereas *χ*^(3)^ displayed a change with one order of magnitude for benzonitrile and carbon disulfide in comparison to the remaining solvents. The non-linear refractive index of PPO samples ranged differently as a function of the solvent characteristics (polarity and molecular symmetry).

### 2.3. Circular Birefringence and Specific Rotation via Channeled Spectra Approach

**Figure 5:**
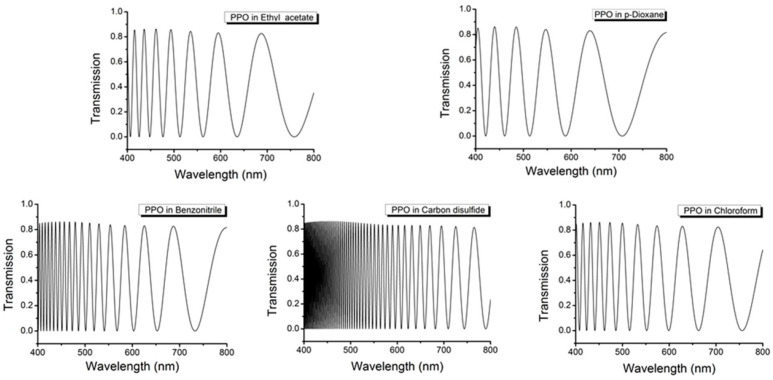
Channeled spectra obtained for PPO in several solvents.

The circular birefringence and optical activity of each computed system were evaluated using the channeled spectrum approach. The benefit of this method is that it facilitates acquiring information on these optical parameters in a wide wavelength interval from a visible range during a single experiment. Figure 5 illustrates the achieved channeled spectra for the PPO–solvent mixtures.

**Figure 6:**
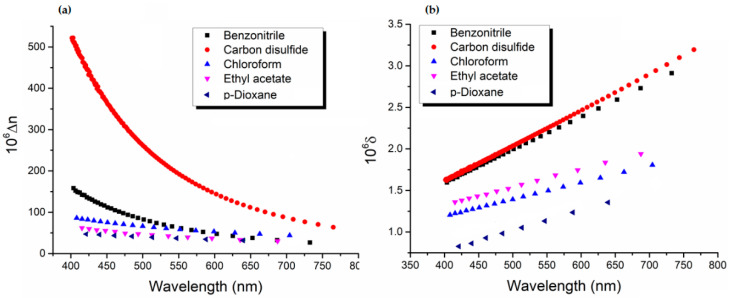
Circular birefringence dispersion (**a**) and birefringence dispersion parameters (**b**) obtained for PPO in several solvents.

**Figure 7:**
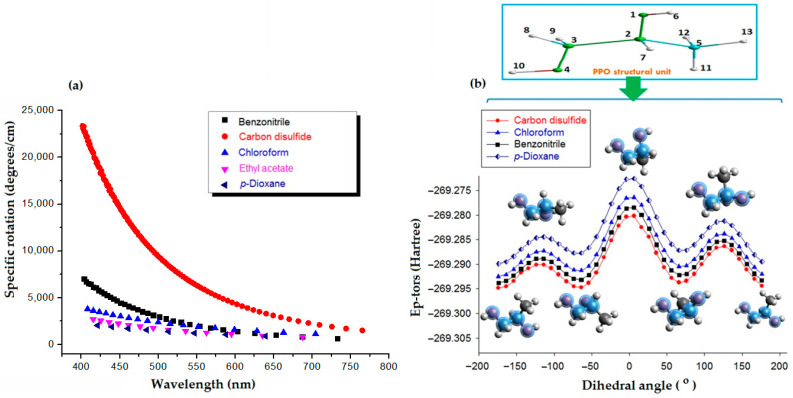
The specific rotation obtained for PPO in several solvents (**a**) and the representation of the optimized configuration of the PPO structural unit with atom order and the torsional potential energy profile versus the O1-C2-C3-O4 dihedral angle from PPO structural unit in the presence of the studied solvents (the numbers from the upper inset index the atoms of the PPO structural unit) (**b**).

The resulting spectra had distinct shapes as a function of the solvent characteristics. When introducing PPO in solvation media characterized by a large polarity and medium hydrogen bonding ability, such as *p*-dioxane and ethyl acetate, the spectrum contained a smaller number of channels, which were wider. On the other hand, solvents characterized by a poor capacity to interact with PPO via hydrogen bonding, like benzonitrile, carbon disulfide, or chloroform, led to spectra with narrow and multiple channels, as noted in Figure 5. Based on the acquired spectral information, the circular birefringence, its dispersion parameter, and specific rotation were evaluated. The resulting data are presented at several wavelengths in Figure 6 and Figure 7.

The circular birefringence is indicative of the level of optical activity of a medium. As seen in Figure 6a, at about 400 nm, the magnitude of Δ*n* of the samples varied depending on the solvent features. Our data revealed that smaller values of Δ*n* were observed for PPO in *p*-dioxane, followed by ethyl acetate solutions. Oppositely, larger values of Δ*n* were noticed for the PPO solutions in carbon disulfide, followed by benzonitrile and chloroform solutions. The achieved circular birefringence results are consistent with other works [46,47,48]. An advantage of the channeled spectra method is that it allows for the estimation of Δ*n* at several wavelengths. As shown in Figure 6a, the Δ*n* dispersion curves show a smaller rate of variation for the solvents characterized by medium hydrogen bonding ability. Conversely, for the solvents with poor predisposition for such interactions, the birefringence dispersion plots have slightly distinct shape with a bigger rate of variation. The dispersion parameter *δ* has a linear increase with the optical radiation wavelength, as seen in Figure 6b. This dependence was nonsignificantly affected by the solvent in which the PPO was introduced. Moreover, the values of the *δ* parameters were small for all analyzed samples.

The channeled spectrum approach also allows for the evaluation of the optical activity of the PPO solutions. The results for specific rotation are displayed in Figure 7a, and they are in agreement with other works employing polarimetry experiments [49,50]. A similar variation of optical rotation with wavelength was reported for PPO dissolved in *p*-dioxane, ethyl acetate, and chloroform [49]. It is known that the specific rotation is directly influenced by the circular birefringence. Thus, analogously to Δ*n*, the optical activity of PPO systems changed when particular solvent features did. As seen in Figure 7a, at about 400 nm, the values of [*θ*] of the PPO solutions were as follows: carbon disulfide > benzonitrile > chloroform > ethyl acetate > *p*-dioxane. Thus, higher values of [*θ*] were noticed for the studied polymer dissolved in carbon disulfide and benzonitrile. As reported in other studies [46,47], the polarity and hydrogen bonding interactions among the solute and solvent molecules caused the modification of the chiroptical response. Also, it is known that the polymer–solvent hydrogen bonding affects the solute rotation in that environment [48]. Therefore, the motional dynamics, assessed via the bond rotational barriers, are limited when the intramolecular interactions in the system components become stronger, so that the probability of rendering different conformations is obviously reduced. The literature [47] additionally shows that, when a solvent tends to interact with the polymer via hydrogen bonding, the value of specific rotation is lower compared to the case when the same polymer is placed in a solvent less able to form such interactions.

Additional insights were acquired by performing molecular modeling to extract the torsional potential energy (*E_p-tors_*) profile for the PPO structural unit in different solvation media (Figure 7b). The *E_p-tors_* profiles versus the dihedral angle were computed by undertaking scans in the range from 0° to 360°, in 10° increments, considering the rotation of the O1-C2-C3-O4 group from the PPO structure. During variation of the torsional angle, distorted and undistorted configurations appeared. Hence, it is possible to detect the dihedral angle interval when the PPO structural unit is most deformed, which is helpful for understanding the distribution of molecular conformations from a thermodynamic point of view. According to the literature [51], the variation in the torsional barrier might be produced by the supplementary steric repulsion generated by the solvent and by hydrogen bonding. In Figure 7b, the data for *E_p-tors_* against the dihedral angle (achieved using the DFT method) are depicted for the PPO structural unit–solvent systems, showing minima and maxima. The lowest values of *E_p-tors_* denote the equilibrium state (high stability of the molecules), while the maxima reflect an unstable state of the molecules (tensioned). The plots from Figure 7b indicate that the PPO structural unit presents one or more conformations in distinct positions that can be ascribed to the smallest energy configuration as a function of the particular solvent features.

The magnitude of the torsional potential energy was higher for the systems where the polar and/or hydrogen boding interactions were stronger (ethyl acetate, *p*-dioxane), thus limiting the free rotation of PPO in these solvation media compared with the other ones. To explain this, one must consider that, in this type of solvent, the chiral substances (like PPO) display more powerful intermolecular forces (e.g., hydrogen bonding, dipole-dipole forces), which raise the barrier to rotation of the substituents from the asymmetric carbon (steep maxima “blocking” rotation). As a consequence, the torsional potential energy has larger values. Given the aforementioned restriction, the PPO structural unit in polar solvents has a narrow possibility to adopt a variety of molecular conformations that can contribute to the overall optical rotation. In other words, linear polarized radiations (presumed to consist of two circularly polarized radiations of opposite sign) passing through the chiral system in polar media will encounter fewer conformers that are able to produce non-symmetric electromagnetic interaction (both circularly polarized beams are slowed down in almost the same manner). This implies a diminished rotation of the light polarization plane, so the expected optical response in such samples is limited. Conversely, if the optically active molecules weakly interact with the solvent, the torsional potential energy will be low (larger mobility of the substituents from the stereogenic center). This favors acquiring a variety of molecular conformations, which enable enhanced non-symmetric electromagnetic interaction (the two circularly polarized rays are differently slowed down), therefore introducing circular birefringence after light exits the chiral medium. As a result, the rotation of the light polarization plane is produced to a larger extent. Thus, molecular modeling supports the specific rotation results, indicating that strong solute–solvent interactions are responsible for diminishing the magnitude of the [*θ*]. The obtained data underline the ability of PPO samples to produce the rotation of the light polarization plane and to modulate it as a function of the solvent nature.

## 3. Materials and Methods

### 3.1. Materials

**Scheme 1:**
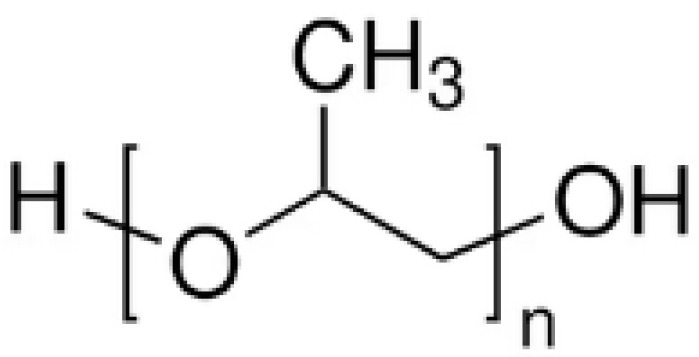
The chemical structure of PPO.

Poly(propylene oxide) (PPO) and the utilized solvents were acquired from Sigma Aldrich (now Merck), St. Louis, MO, USA. The chemical structure of the polymer is displayed in Scheme 1.

The polymer solutions were prepared by dissolving 2.5 g of PPO powder in 100 mL of each of the following solvents, acquired from Sigma Aldrich (now Merck): benzonitrile (anhydrous, ≥99%), carbon disulfide (anhydrous, ≥99%), chloroform (anhydrous, ≥99%, contained 0.5–1.0% ethanol as a stabilizer), ethyl acetate (anhydrous, ≥99.8%), and *p*-dioxane (anhydrous, ≥99.8%). The pH of the polymer solutions did not vary intentionally during experiments, and the small variations were caused by the combination of PPO (pH = 4) with the selected solvents having pH values comprised between 4 and 7.

### 3.2. Characterization

The rheological behavior of the PPO samples was analyzed on the Bohlin CS50 device (Malvern Instruments Ltd., Malvern, UK). The employed measuring system had a cone/plate geometry (4 cm/4°) with a gap of 150 µm. Shear viscosities were registered over a 0.1–100 s^−1^ shear rate range (at 25 °C), and for evaluation of the activation energy, the temperature varied between 25 and 45 °C. Shear moduli were measured within the linear viscoelastic regime of the samples, where the rheological moduli were not affected by the strain amplitude. The frequency sweep experiments involved an application of 0.1–50 Hz and a stress of 1 Pa.

The refractometry testing of each PPO system was carried out at 25 °C on multi-wavelength Abbe equipment (Anton Paar GmbH, Graz, Austria) having a precision of 10^−4^.

The molecular modeling was carried out with Gaussian G16 software (Gaussian, Inc., Wallingford, CT, USA) [52]. The density functional theory (DFT) approach was used for the proposed computations. The PBE0/6-31+G(d,p) method was employed for optimization and single point calculations. The scanning of the torsion angle corresponding to the atoms O1-C2-C3-O4 from the PPO repeating unit was performed to search the conformational landscape. This was done by taking into account the dihedral angle and including the solvent effect (implicit model). After attaining the global minimum conformation, two solvent molecules were added in the presence of PPO to test the possible occurrence of the specific interactions (e.g., hydrogen bonding). The solute–solvent pair was reoptimized to acquire equilibrium of the molecular system in an implicit solvent model. Then, the recalculation of the Hessian matrix (frequency calculation) was done to check if the negative frequencies were lacking and thus confirming the equilibrium state of the system. Based on theoretical analyses, the distance corresponding to hydrogen bonding was measured to be smaller than 3 Å. The functional PBE0 [53] can predict reliable results relating to the intramolecular/intermolecular structural parameters as well as about the conformational effect in organic compounds [54,55,56]. The 6-31+G(d,p) basis set employed together with a functional method reported a good estimation of wave functions of the optimized structures [57].

The channeled spectra of all solutions were recorded at ambient temperature (25 °C) using a UV–VIS spectrophotometer (Carl Zeiss Jena, Jena, Germany). The device had a data acquisition system, which enabled the registering of spectra with a resolution of 0.2 nm. A transparent cell of 2.5 dm in size was employed for the measurements. The experimental setup was described in detail in another work [28]. Briefly, the double beam spectrophotometer operated using linear polarized light, which was produced by placing polarizers in the device along the sample beam and along the reference beam. More precisely, the polymer solution cell was inserted in the measurement beam between two polarizers with crossed transmission directions, while in the reference beam, the polarizers had parallel orientation (due to absorption compensation). By this procedure, the linear polarized light passed through the chiral polymer solution, and its ordinary and extraordinary components underwent interference. Thus, the recorded spectral signal intensity presented a periodic modulation function of the wavelength. The channeled spectrum was attained with a speed of 2 cm^−1^/s. In this way, it was possible to discern the changes in the flux density from a channel to the neighboring one.

### 3.3. Theoretical Background

The rotation angle of the polarization plane was affected by several parameters, as depicted in Equation (9):

(9) $$\theta =\left[\theta \right]CL=\frac{\pi }{\lambda_{0}}\Delta n\;CL$$

where [*θ*] is the specific rotation, *C* is the concentration, *L* is the length of anisotropic medium, *λ*_0_ is the radiation wavelength in a vacuum, and Δ*n* is the rotatory birefringence.

The polarization plane remains unchanged or spins with an integer of π in the situation in which the radiation displays the electric field orthogonal to the polarizer transmission direction, generating a null flux density. On the other hand, when the polarization plane is changed upon spinning with an odd number of π/2, the radiations display the electric field parallel to the polarizer transmission direction, producing the biggest flux density (channeled spectrum presents maxima).

The transmission factor of the spectrophotometer, denoted here as *T*, is defined in Equation (10):

(10) $$T=\sin^{2}\frac{\pi }{\lambda_{0}}\Delta n\;CL$$

The spectrophotometer uses a quasi-equienergetic source of optical radiation, which passes through the polymer solution, and the emergent beams have components of variable degrees of rotation.

The wavenumber data ascribed to minima (of order *k*) and maxima (of order *k* + 1/2) of the channeled spectra can be employed to evaluate the circular birefringence; its dispersion parameter, denoted as *δ*; and order of the channels, denoted as *k*. The results leading to the calculation formulae were detailed in previous works [28] and are shown in Equations (11)–(13):

(11) $$\lambda_{0,k}=\frac{(\Delta n-\delta )CL}{k};\;k=1,\;2,\;3,\ldots$$

(12) $$\lambda_{0,k+1/2}=\frac{2\Delta nCL}{2k+1};\;k=0,\;1,\;2,\;3,\ldots$$

(13) $$\lambda_{0,k+1}=\frac{(\Delta n+\delta )CL}{k+1};\;k=0,\;1,\;2,\;3,\ldots$$

Equations of the type (11)–(13) are useful for obtaining Δ*n*, *δ*, and *k* parameters for each PPO–solvent system at various wavelengths based on Equations (14)–(16):

(14) $$\Delta n=\frac{1}{2L}\frac{\lambda_{0k+1/2}(\lambda_{0k}-\lambda_{0k+1})}{\lambda_{0k+1}-2\lambda_{0k+1/2}+\lambda_{0k}}$$

(15) $$\delta =\frac{1}{2L}\frac{2\lambda_{0k}\lambda_{0k+1}-\lambda_{0k+1/2}\lambda_{0k}-\lambda_{0k+1}\lambda_{0k+1/2}}{\lambda_{0k+1}-2\lambda_{0k+1/2}+\lambda_{0k}}$$

(16) $$k=\frac{1}{2}\frac{\lambda_{0k+1}-\lambda_{0k+1/2}}{\lambda_{0k+1}-2\lambda_{0k+1/2}+\lambda_{0k}}$$

## 4. Conclusions

This work describes the effects of several solvents on the optical activity of PPO solutions. Rheological analyses revealed that in low polar solvents, the interactions of PPO systems were weaker, leading to lower flow activation energy and a higher interaction solute–solvent radius. Molecular modeling emphasized the sites from the PPO structure that are available for the formation of the hydrogen bonds in the presence of certain solvents. The prepared polymer solutions exhibited normal dispersion curves, and the recorded refractivity was affected by the solvent’s optical features. The linear optical parameters like dispersion energy and band gap energy presented lower values for samples in more polar solvents. Also, the first-order optical susceptibility and non-linear refractive index were slightly smaller for PPO in such solvents. The data attained from the channeled spectrum enabled evaluation of the circular birefringence, its dispersion parameter, and specific rotation. At about 400 nm, the circular birefringence was higher for the systems where the solvent had low polarity and a poor ability to connect via hydrogen bonds with PPO. The dispersion parameter of birefringence displayed small values, while the circular birefringence dispersion curves had slightly different shapes for the systems prepared in polar solvents having a medium capacity to form hydrogen bonds with the polymer. Similar variation was noted for specific rotation of the samples, which was found to range in the next order: carbon disulfide > benzonitrile > chloroform > ethyl acetate > *p*-dioxane. This was sustained by molecular modeling, which showed that there is a correlation between the solvent nature, the rotational barrier of the substituents of asymmetric carbon from PPO, and the optical response. The resulting powerful interactions among the chiral molecules and the solvent limited the mobility of substituents from the stereogenic center and thereby restricted the number of conformers that can generate non-symmetric electromagnetic interaction; consequently, the specific rotation was diminished.

As a conclusion, this investigation emphasized the role of polymer–solvent interactions on tailoring optical properties, including the optical activity parameter, which has practical implications for designing supramolecular architectures for photonic or biomedical devices.

## Figures and Tables

**Table 1:** The values of the flow behavior index and consistency index for PPO in several solvents.

System	Flow Behavior Index	Consistency Index (Pa.s^n^)
PPO/*p*-dioxane	0.42	0.48
PPO/Ethyl acetate	0.39	0.19
PPO/Chloroform	0.81	0.07
PPO/Carbon disulfide	0.73	0.02
PPO/Benzonitrile	0.44	0.34

**Table 2:** The data of the dispersion energy (*E_d_*), single-oscillator energy (*E*_0_), band gap energy (*E_g_*), static refractive index (*n*_0_), first-order optical susceptibility (*χ*^(1)^), third-order optical susceptibility (*χ*^(3)^), and non-linear refractive index (*n_nl_*) of the PPO solutions in several solvents.

System	*E_d_* (eV)	*E*_0_ (eV)	*E_g_* (eV)	*n_0_*	*χ* ^(1)^	*χ*^(3)^ (e.s.u.)	*n_nl_* (e.s.u.)
PPO/*p*-dioxane	6.005	6.455	3.228	1.389	0.074	5.107 × 10^−15^	1.385 × 10^−13^
PPO/Ethyl acetate	5.924	7.339	3.669	1.344	0.064	2.894 × 10^−15^	8.118 × 10^−14^
PPO/Chloroform	10.957	10.529	5.265	1.428	0.082	7.996 × 10^−15^	2.109 × 10^−13^
PPO/Carbon disulfide	14.899	9.699	4.849	1.592	0.122	3.797 × 10^−14^	8.984 × 10^−13^
PPO/Benzonitrile	9.578	8.156	4.078	1.476	0.093	1.297 × 10^−14^	3.315 × 10^−13^

## Data Availability

The data presented in this study are available on reasonable request from the corresponding author.
